# First person – Sarit Pal

**DOI:** 10.1242/bio.059490

**Published:** 2022-07-25

## Abstract

First Person is a series of interviews with the first authors of a selection of papers published in Biology Open, helping early-career researchers promote themselves alongside their papers. Sarit Pal is first author on ‘ Nuclear localization of histamine receptor 2 in primary human lymphatic endothelial cells’, published in BiO. Sarit conducted the research described in this article while a graduate research assistant in Dr Anatoliy Gashev's lab at Texas A&M University, Bryan, TX, USA. He is now a senior field application scientist at BICO LLC, Waltham, MA, USA, investigating histamine signalling in the regulation of lymphatic immunophysiology.


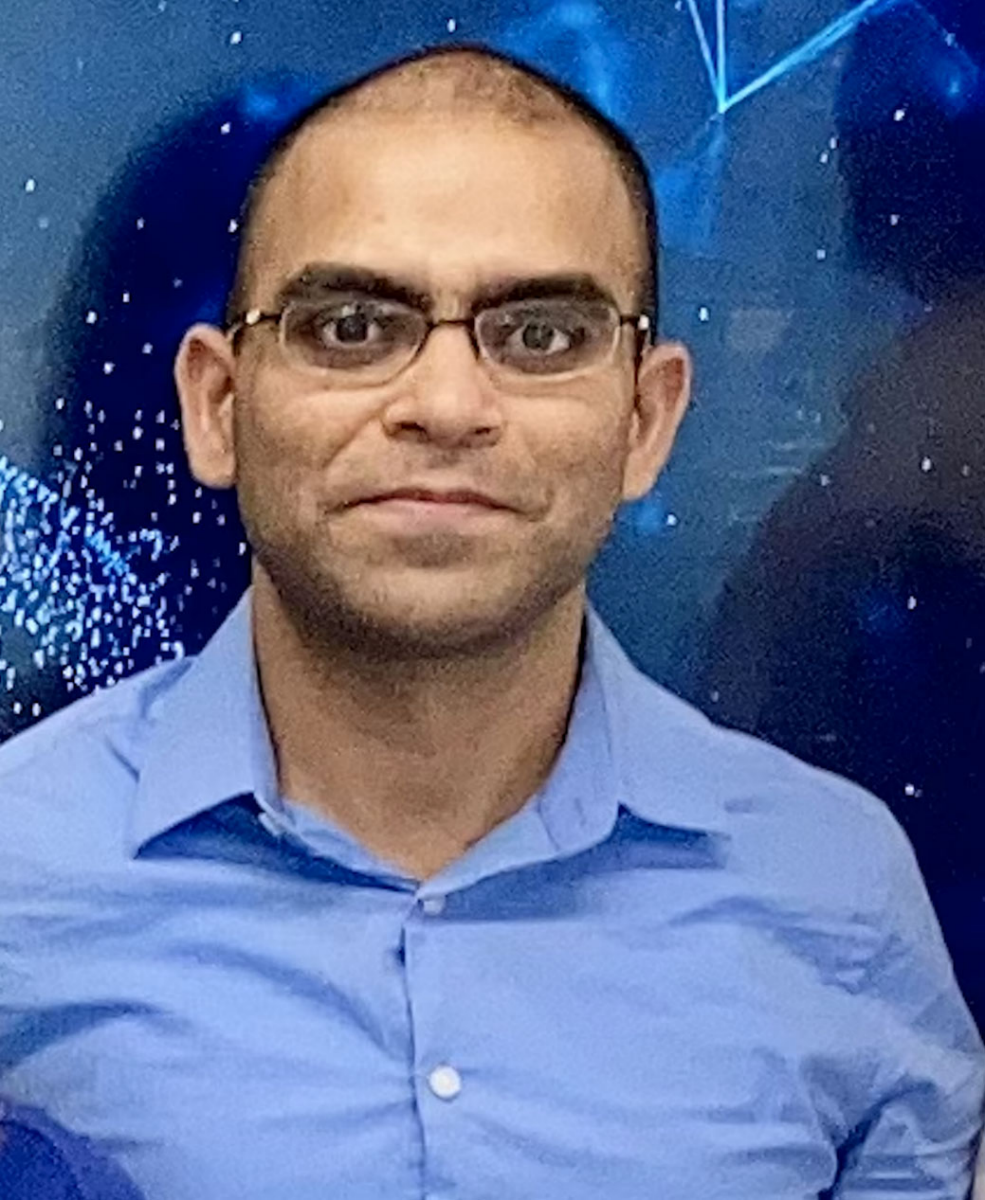


**Sarit Pal**

**What is your scientific background and the general focus of your lab?**

I started my career as a veterinarian; however, initial exposure to research during my DVM inspired me to pursue a career in biomedical research. I pursued my MS thesis in chemistry in self-assembly-based DNA delivery systems, and my doctoral thesis was focused on understanding how histamine signalling regulates lymphatic immunophysiology and influence intersystem crosstalk. Being fascinated with the lymphatic system, during my postdoc I studied organ-specific lymphatic vasculature, such as in the cerebral nervous system and liver. Almost any organ in our body has lymphatic circulation, but our understanding of their contributions in maintaining homeostasis or in disease progression is far from complete. In the future, I am interested in exploring the development of bioprintable lymphatic vasculature that are capable of grafting, as well as developing *in vitro* organ models with lymphatic vessel innervation for studying specific diseases.

“Our findings […] will not only broaden the scope of H2R biology but also shed light on unexplored H2R-related pathophysiologies.”

**How would you explain the main findings of your paper to non-scientific family and friends?**

Histamine receptor 2 (H2R) blockers, such as ranitidine and cimetidine, are commonly used over-the-counter drugs. However, chronic ingestion of these H2R blockers has recently been shown to have adverse effects, such as association with gastrointestinal cancer. In addition, H2R is expressed in multiple tissue types. Interestingly, our present understanding of H2R biology, its structure–function relationship and signalling mechanism is far from complete. In this study, we show a unique nuclear localization pattern of H2R. Multiple hormone receptors, such as oestrogen and beta 1 adrenergic receptor, were shown to be localized in the nucleus as a G protein-coupled receptor and involved in gene expression and downstream signalling cascades. Our findings of H2R's nuclear localization thus indicate that H2R can potentially be involved in such functions, which will not only broaden the scope of H2R biology but also shed light on unexplored H2R-related pathophysiologies.

**What are the potential implications of these results for your field of research?**

In future study, if we can elucidate the functional implication of such H2R nuclear localization – for example, if H2R contributes towards the transcription of specific genes – that can open up new possibilities of H2R as a therapeutic target. Furthermore, histamine is a ubiquitously expressed molecule; in the presence of histamine, if H2R can influence transcription of a lymphatic vasculature-specific set of genes, this could be a significant piece of information in the lymphatic field.

**Figure:**
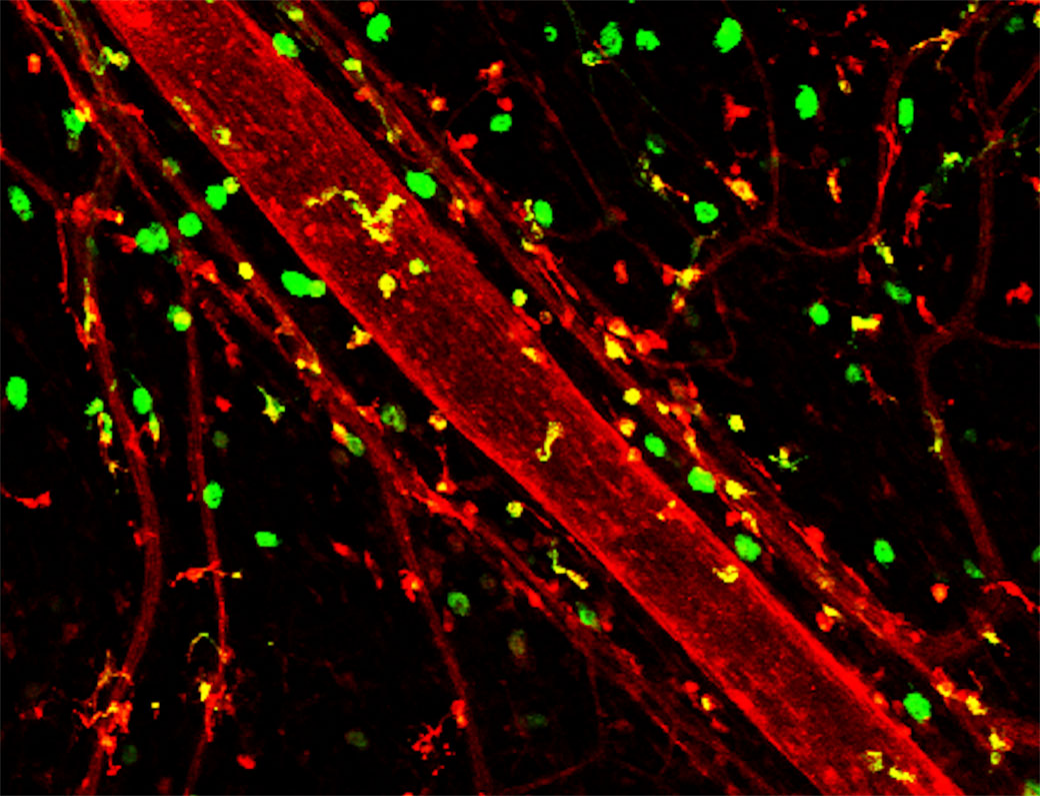
**The close association of lymphatic vasculature with immune cells.** Green, MHCII-positive cells; red, histamine receptor 1.

**What has surprised you the most while conducting your research?**

To maintain homeostasis or in response to its perturbation, different physiological systems (such as vascular, nervous, immune) in our body need to communicate. I always wondered what are the bases of these intersystem communications/crosstalks? What are the messenger molecules, or their receptors, which are ubiquitously expressed in multiple tissue types?

**What, in your opinion, are some of the greatest achievements in your field and how has this influenced your research?**

Lymphatic research in the last 10 years has seen remarkable progress. People from multiple disciplines have started exploring lymphatics. Now we have a basic understanding of multiple organ-level lymphatic vasculatures such as the cerebral nervous system, kidney, lung and liver, and the association of the lymphatic circulation with immunity and inflammation. The lymphatic system is not just a passive drainage system, it is as important as the blood vascular system. Especially discoveries in organ-level lymphatic circulation have inspired my postdoctoral works and future work.

**What changes do you think could improve the professional lives of early-career scientists?**

Having faith in creative, motivated early-career individuals, being unbiased towards their diverse point of views and their diverse educational backgrounds as well. Also, creating a safety net that will allow early-career scientists to take risks to pursue bold ideas and take chances, even if they know there is a good possibility of failure.

“Life is full of surprises like doing science.”

**What's next for you?**

Who knows? Life is full of surprises like doing science. Rather than planning my next step, I always like the idea of enjoying what I am doing now passionately, then making a decision and moving forward as it comes.
